# Development of antibodies to human embryonic stem cell antigens

**DOI:** 10.1186/1471-213X-5-26

**Published:** 2005-11-29

**Authors:** Jingli Cai, Judith M Olson, Mahendra S Rao, Marisa Stanley, Eva Taylor, Hsiao-Tzu Ni

**Affiliations:** 1 Stem Cell Biology Unit, Laboratory of Neurosciences, National Institute on Aging, 333 Cassell Dr, Rm406A, Baltimore, MD 21224, USA; 2 Stem Cell Department, R&D Systems, Inc., 614 McKinley Place. Minneapolis, MN 55413, USA

## Abstract

**Background:**

Using antibodies to specific protein antigens is the method of choice to assign and identify cell lineage through simultaneous analysis of surface molecules and intracellular markers. Embryonic stem cell research can be benefited from using antibodies specific to transcriptional factors/markers that contribute to the "stemness" phenotype or critical for cell lineage.

**Results:**

In this report, we have developed and validated antibodies (either monoclonal or polyclonal) specific to human embryonic stem cell antigens and early differentiation transcriptional factors/markers that are critical for cell differentiation into definite lineage.

**Conclusion:**

These antibodies enable stem cell biologists to conveniently identify stem cell characteristics and to quantitatively assess differentiation.

## Background

Although the stem cell concept was introduced decades ago, to date, stem cells can only be defined functionally, not morphologically or phenotypically. Two functions define stem cells. Firstly, they are self-renewing, thus able to propagate to generate additional stem cells. Secondly they can differentiate into various progenitor cells, which commit to further maturation along a specific lineage. While stem cells can be best defined functionally, a good number of molecular markers have been used to prospectively identify various stem cell populations. Although the functional importance of many of these antigens remains unknown, their unique expression pattern and timing of expression provide a useful tool for scientists to identify as well as isolate stem cells.

Embryonic stem cells (ESC), derived from the inner cell mass of pre-implantation embryos, have been recognized as the earliest stem cell population [1,2]. This pluripotent population can differentiate into all somatic tissue including germ cells. In the case of human ESC, they can differentiate into some extra-embryonic derivatives as well. Like mouse ESC, human ES cells can be maintained and propagated on mouse fibroblast feeders for extended periods in media containing basic fibroblast growth factor (bFGF) [3]. Gene expression of undifferentiated human ES cells has been investigated among several ES cell lines by a variety of techniques. They include comparison with databases, reverse transcriptase-polymerase chain reaction, focused cDNA microarrays, and immunocytochemistry. A list of molecules comprised of known ES-specific or -highly expressed genes and candidates that can serve as markers for human ESCs and may also contribute to the "stemness" phenotype has been established [3-11]. For example, pluripotent ESC can be characterized by high level expression of Oct3/4 (POU domain, class 5, transcription factor 1, Pou5f1) and Nanog, which are a member of POU domain and homeobox transcription factors respectively. A critical amount of Oct3/4 and Nanog expression is required to sustain stem-cell pluripotency and both of these markers are downregulated as cells differentiate in vitro and in vivo [4-9]. Antibodies to Oct3/4 which cross react with human Oct 3/4 have been widely used to monitor the presence of undifferentiated ESC.

No single marker however is sufficient or unique for identifying ESCs. Oct3/4 for example is expressed by germ cells and may be expressed by specific populations later in development. Likewise, Nanog has been shown to express in other tissues. We and other have noted however, that while no single marker is sufficient a constellation of positive and negative markers can in concert unambiguously allow one to define the state of ESC cultures and that surface markers in combination can be used to prospectively sort for ESC.

Based on published data at the level of gene expression, we have cloned a number of candidate marker genes. We have also expressed the recombinant protein and generated a panel of monoclonal or polyclonal antibodies to these proteins. Using these antibodies we have confirmed the specificity and selectivity of these antibodies on several ESC lines and established their utility as stem cells markers. Our results confirm the expression pattern and timing of these cell markers at the protein level, whereas previous data reported at the level of gene expression.

## Results and discussion

### Characterization of undifferentiated human ES cells and differentiated EBs by antibodies

All monoclonal antibodies were initially selected for their abilities to recognize recombinant proteins in direct ELISAs. A subset were also tested by Western Blot analysis using recombinant proteins and cell lysate to confirm binding to a single epitope. The best clone was later screened for its applications for immunocytochemistry and flow cytometry using various cell lines. Human peripheral blood platelets were used for screening mouse anti-human CD9 antibody. MCF-7 cells were used for screening mouse anti-human E-Cadherin and PODXL (podocalyxin-like) antibodies. MG-63 cells were used for screening mouse anti-human GATA1 (GATA binding protein 1) antibody. Beta-TC6 cells were used for screening for mouse anti-human/mouse PDX-1 (pancreatic duodenal homeobox-1) antibody. NTERA-2 cells were used for screening mouse anti-human Oct3/4 and SOX2 (sex-determining region Y-box 2) antibodies. All polyclonal antibodies were affinity-purified using recombinant proteins and validated by direct ELISAs and Western. Caco-2 cells were used for validation of goat anti-human GATA6 antibody and NTERA-2 cells were used for validation of goat anti-human Nanog and anti-human Oct3/4 antibodies (Summarized in Table 1).

**Table 1:** Summary list of antibody verification by western blot.

**Antibody**	**Sample used for analysis**	**Mol. Wt. (KD)**
Gt × hBrachyury	mouse ES-derived EB lysate	48
Ms × hDPPA5	N/A	N/A
Gt × hGATA6	Caco2 cell lysate	65
Gt × hNanog	NTERA-2 cell lysate	33
Gt × hOct 3/4	NTERA-2 cell lysate	39
Gt × hPDX1	beta-TC 6 cell lysate	32
Gt × hSOX17	mouse ES-derived EB lysate	45
Ms × hCD9	PBMC	25
Rt × hGATA-1	N/A	N/A
Ms × hE-Cadherin	MCF-7 cell lysate	97
Ms × hPODXL	MCF-7 cell lysate	57
Ms × hSOX2	NTERA-2 cell lysate	36

N/A: 1. DPPA5 is still being subcloned. Only Elisa verification is available.

2. The clone for GATA-1 (MAB1779) does not work for Western blot application but is useful for IHC, The clone picked for Western blot analysis does not work for IHC (MAB17791, see data in ).

**Figure 1:**
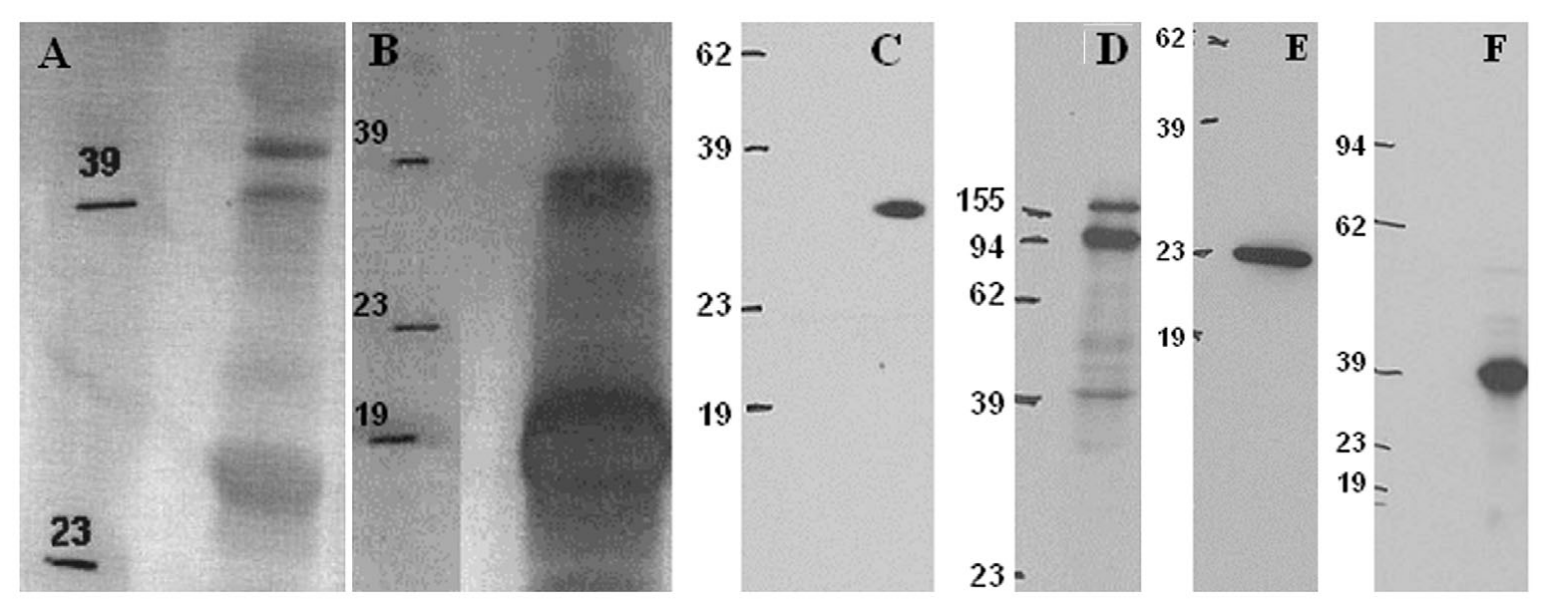
Western blot analysis for Gt × hOct3/4 (A), Gt × hNanog (B) and Ms × hSOX2 (C) in NTERA-2 cell lysate, Ms × hE-Cadherin (D) in MCF-7 cell lysate, Ms × hCD9 (E) in PBMC lysate and Ms × hPDX-1(F) in β-TC-6 cell lysate. Numbers indicate the positions of molecular weight markers.

**Figure 2:**
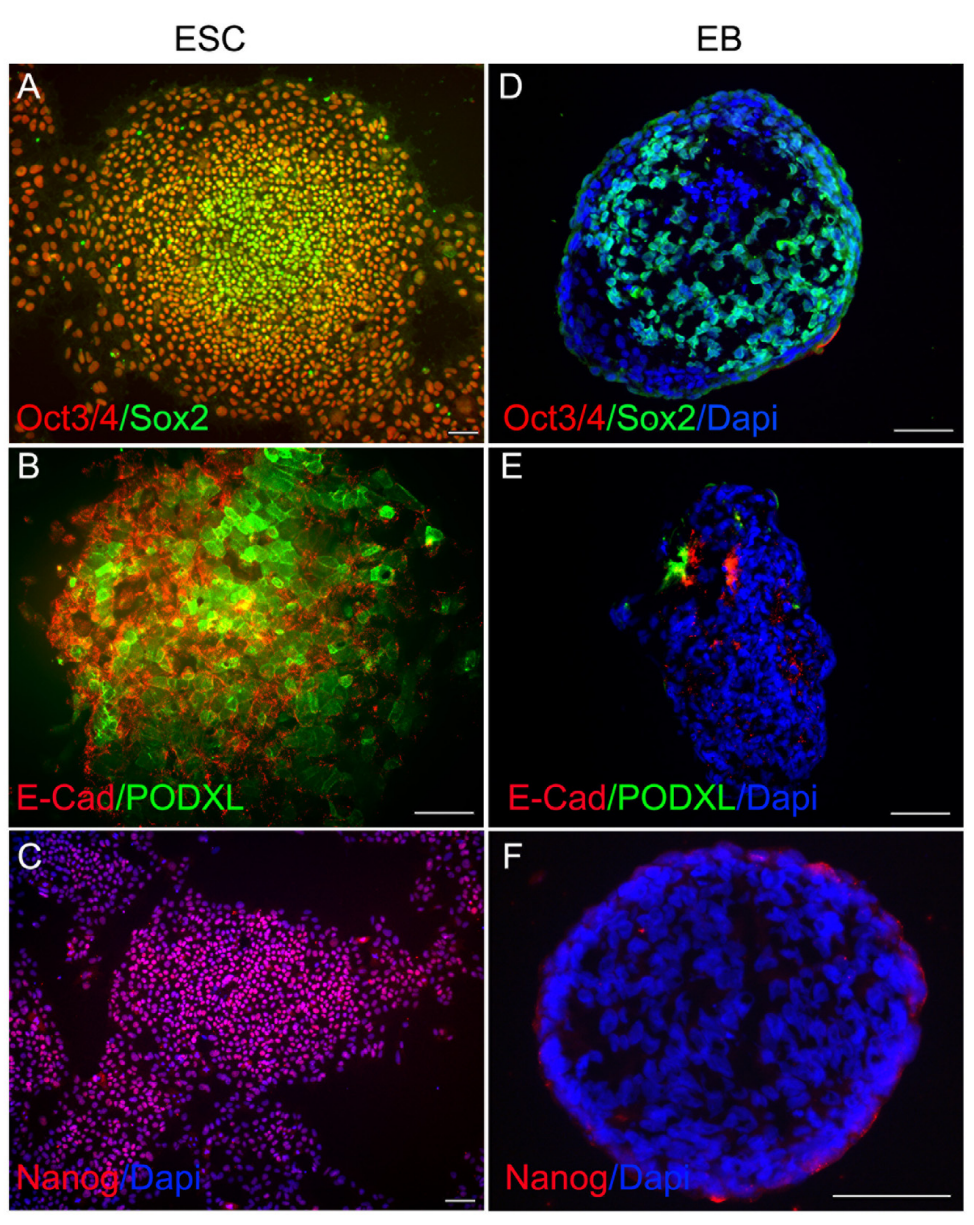
Undifferentiated human ES cells (A, B, and C) and differentiated EBs (D, E and F) were analyzed using antibodies to indicated molecular markers. Immunostaining with goat anti-human Oct3/4 (Red in A and D), mouse anti-human SOX2 (Green in A and D), goat anti-human E-Cadherin (Red in B and E), mouse anti-human PODXL (Green in B and E), and goat anti-human Nanog (Red in C and F), are contrasted with DAPI nuclear staining (Blue in C-F). Note the dramatic downregulation of ESC specific markers (Oct3/4, E-Cadherin, PODXL, and Nanog) in EBs. However, SOX2 expression is persistent in subsets of EB cells. Scale bars = 100 μm.

**Figure 3:**
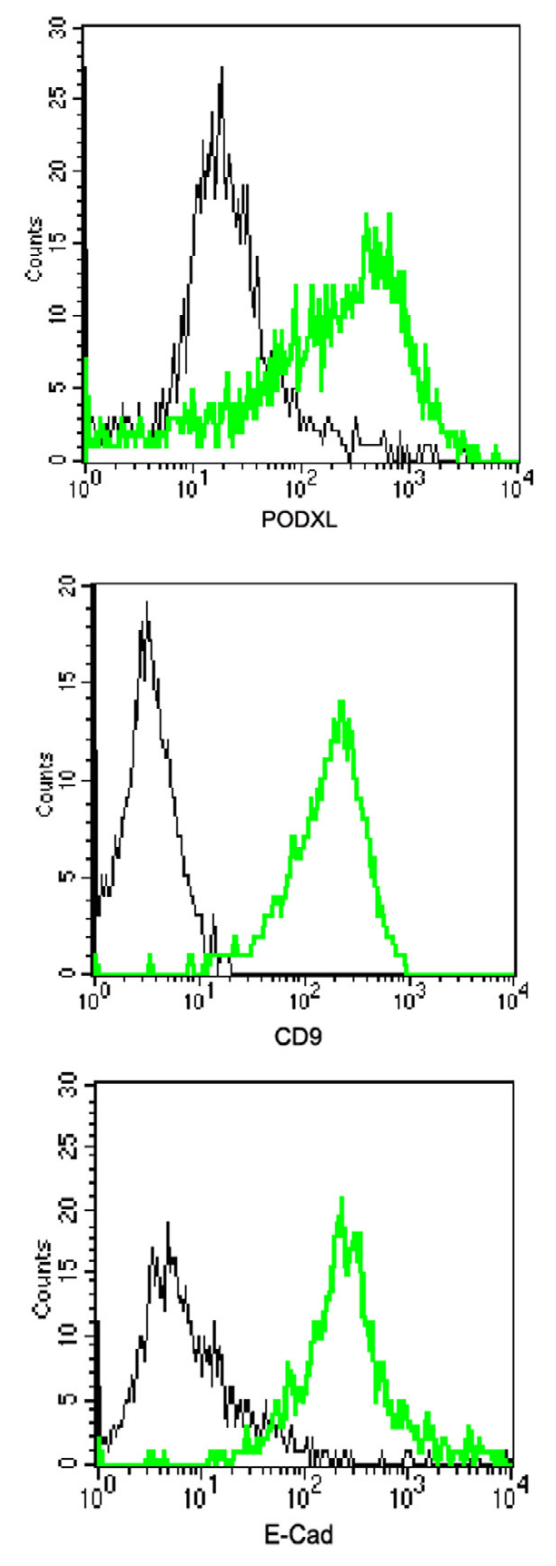
Human embryonic stem cells stained with anti-CD9 (A), anti-E-Cadherin (B), and anti-PODXL (C) and antigen expression detected by a flow cytometer. The specific staining is indicated by green histogram and corresponding isotype control is indicated by black histogram.

After antibodies were validated in direct ELISAs, Western blot or cell lines (Fig. 1 and data not shown), they were used to examine the expression of individual molecules in undifferentiated human ES cells and differentiated EBs. When examined by immunohistochemistry, high level of expressions of Oct3/4, SOX2, E-Cadherin, PODXL and Nanog were observed in undifferentiated human ES cells (Fig. 2A, 2B and 2C). DPPA5 (developmental pluripotency associated 5) expression was also observed in undifferentiated human ES cells (data not shown). We noted that a subset of the proteins used were membrane bound proteins. To test if any of the antibodies generated could recognize an extracellular epitope and thus be used for live cell sorting, we repeated staining of live cells as previously described. The CD9, E-Cadherin and PODXL antibodies recognized an extracellular epitope and their ability to select cells by FACS was confirmed (Fig. 3). Minimal or no expressions of Oct3/4, E-Cadherin, PODXL and Nanog were detected in the differentiated EBs (Fig. 2D, 2E and 2F). However, SOX2 expression, which is observed in neural progenitor cells, is persistent in subsets of EBs.

**Figure 4:**
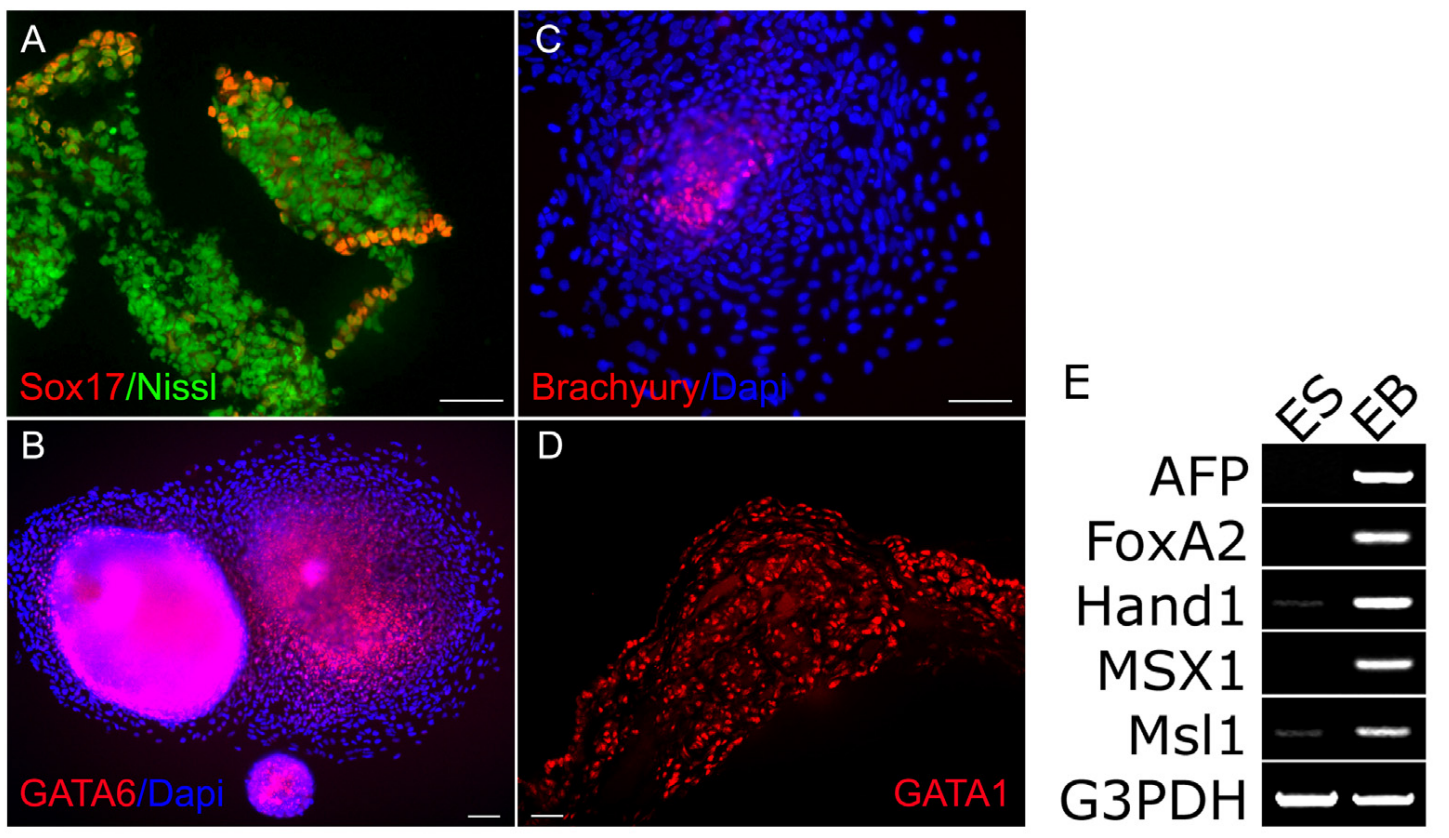
Differentiated EBs were analyzed by either immunocytochemistry or RT-PCR to the indicated molecular markers. (A) Immunostaining with goat anti-human SOX17 (Red), is contrasted with Fluoro Nissl nuclear staining (Green). (B) Immunostaining with goat anti-human GATA6 (Red), is contrasted with DAPI nuclear staining (Blue). (C) Immunostaining with goat anti-human brachyury (Red), is contrasted with DAPI nuclear staining (Blue). (D) Immunostaining with mouse anti-human GATA1 (Red). Note that each antibody recognizes subsets of EB cells. Scale bars = 100 μm. (E) The differentiation status of EB is detected by RT-PCR using different germ layer cell markers. Selected endoderm markers AFP, FoxA2; mesoderm markers Hand1, MSX1 and ectoderm marker Msl1 were all highly expressed in the EB samples while their expression was either undetectable or at low level in the ES samples. G3PDH was a positive control showing similar amount of RNA samples were used for analysis.

Suspension culture with FGF withdrawal is known to induce differentiation of ES cells to all three germ layer precursors [12]. The differentiation status of the EB used here was detected to contain all germ cell markers by RT-PCR (Fig. 4). In order to examine how more antibodies can be used for characterization of early differentiation events from human ES cells, we examined the expressions of endodermal markers, SOX17, GATA6 and PDX-1, and mesodermal markers, Brachyury and GATA1, in the undifferentiated human ES cells and differentiated EBs. Expressions of SOX17, GATA6, PDX-1, Brachyury and GATA1 were not detected in undifferentiated human ES cells (data not shown). In contrast to the undifferentiated ES cells, subpopulations of SOX17-, GATA6-, Brachyury- and GATA1-positive cells were observed (Fig 4). These results suggest that both endodermal and mesodermal precursors exist in EBs with FGF withdrawal for 8 days. However, no PDX-1-positive cells were seen in EBs differentiated with the same treatment (data not shown).

### Examination of cross-reactivity of antibodies on mouse ES and differentiated cells

We have also examined the cross-reactivities of these antibodies to mouse ES cells using mouse D3 ES cell line and mouse fetal endodermal tissue. Cross-reactivity to mouse of goat anti-Oct3/4, goat anti-PDX-1, goat anti-SOX17 and mouse anti-SOX2 was detected. Minimal cross-reactivity to mouse, measured by 10% intensity to human by higher than control cells, was observed in mouse anti-CD9 and mouse anti-E-cadherin antibodies. Goat anti-Nanog and mouse anti-PODXL antibodies appear to be human-specific as well (data not shown). The subtypes of monoclonal antibodies were also identified in the best clones. These results are summarized in Table 2.

**Table 2:** Summary of antibodies detection in ES and EB samples.

**Antibody**	**ES**	**EB**	**Reactivity to mouse**	**Isotype of monoclonal antibody (Clone No.)**
**Gt × hBrachyury**	No	Yes	NT*	
**Ms × hDPPA5**	Yes	NT*	NT*	ND*
**Gt × hGATA6**	No	Yes	NT*	
**Gt × hNanog**	Yes	Down	No	
**Gt × hOct 3/4**	Yes	Down	Yes	
**Gt × hPDX-1**	No	No	Yes	
**Gt × hSOX17**	No	Yes	Yes	
**Ms × hCD9**	Yes	No	Minimal	Mouse IgG2B (clone 209306)
**Ms × hE-cadherin**	Yes	No	Minimal	Mouse IgG2B (clone 180224)
**Ms × hGATA1**	No	Yes	NT*	Rat IgG2B (clone 234732)
**Ms × hPODXL**	Yes	No	No	Mouse IgG2A (clone 222328)
**Ms × hSOX2**	Yes	Yes	Yes	Mouse IgG2A (clone 245610)

*NT, Not tested; ND, Not determined.

## Conclusion

The expression patterns detected using antibodies developed in our facility are consistent with data reported using reverse transcriptase-polymerase chain reaction or cDNA microarrays. Moreover several of the monoclonal antibodies have differing heavy chain subunits allowing double labeling using subtype specific markers to be performed.

In summary, we have developed a useful collection of antibodies that would be useful for identification of stem cell characteristics and assessment of differentiation. Several additional antibodies to the molecules that have been identified as potential cell lineage markers [13] are currently under development using the same approach.

## Methods

### Cloning and expression of Brachyury, DPPA5, CD9, E-Cadherin, GATA1, GATA6, Nanog, Oct3/4, PDX-1, PODXL, SOX2 and SOX17

Brachyury (aa. 1–202), DPPA5 (a.a. 1–116), GATA1 (a.a. 1–413), GATA6 (aa. 1–449), Nanog (aa. 153–305), Oct3/4 (aa. 1–265), PDX-1 (aa. 1–283), SOX2 (aa. 135–317) and SOX17 (aa. 177–414) were expressed in E. Coli and extracellular domains of CD9, E-Cadherin, PODXL were expressed in mouse NSO cells. All proteins were purified and sequenced before they were used as antigens for immunizations and as substrate for antibody screening and subcloning.

### Production and purification of antibodies

All monoclonal antibodies were derived from fusions of mouse myeloma with B cells obtained from BALB/c mice which had been immunized with purified antigen. The IgG fraction of the culture supernatant was purified by Protein G affinity chromatography (Sigma). Each panel of antibodies was screened and selected for their abilities to detect purified recombinant antigen in direct ELISA and Western blot. All polyclonal antibodies were derived from sera of goats which had been immunized and boost it with purified antigen. Antibody was purified from the sera by an antigen-affinity chromatography.

### Cells and cell culture

Human Caco-2, MG-63, MCF-7, NTERA-2 and mouse D3 cells were purchased from American Type Culture Collection (ATCC). Cells were cultured according to the ATCC instructions. Information regarding human ES cell line HSF-6 (NIH code UC06) can be obtained at the website [14]. Undifferentiated human ES cells were cultured according to the protocol provided by the University of California, San Francisco in human ES culture medium [DMEM supplemented with 20% KnockOut Serum Replacement (Invitrogen) and 5 ng/mL of bFGF (R&D Systems)]. To induce formation of embryoid bodies (EBs), ES colonies were harvested, separated from the MEF feeder cells by gravity, gently resuspended in ES culture medium and transferred to non-adherent suspension culture dishes (Corning). Unless otherwise noted, EBs derived from human ES cell aggregates were cultured for 8 days in ES culture medium deprived of bFGF and used for analysis by immunohistochemistry as described.

### Western blot

Cells are solubilized in hot 2× SDS gel sample buffer (20 mM dithiothreitol, 6% SDS, 0.25 M Tris, pH 6.8, 10% glycerol, 10 mM NaF and bromophenyl blue) at 2 × 10^6 ^per mL. The extracts are heated in a boiling water bath for 5 minutes and sonicated with a probe sonicator with 3–4 bursts of 5–10 seconds each. Samples are diluted with 1× SDS sample buffer to the desired loading of 1–5 × 10^3 ^per lane. Lysates were resolved by SDS-PAGE, transferred to Immobilon-P membrane, and immunoblotted with 0.5 μg/mL primary Abs as described in R&D Systems Website [15].

### Immunohistochemistry

Antibodies were used with the appropriate secondary reagents at a concentration of 5 to 10 μg/ml. Cells or sections of EBs were fixed with 4% paraformaldehyde in PBS at room temperature for 20 min, then blocked and permeabilized with 0.1% Triton X-100, 1% BSA, 10% normal donkey serum in PBS at room temperature for 45 min. After blocking, cells were incubated with diluted primary antibody overnight at 4°C followed by coupled anti-mouse or anti-goat IgG (Molecular Probes) at room temperature in the dark for an hour. Between each step cells were washed with PBS with 0.1% BSA.

### RT-PCR

Total RNA was extracted from EBs using Trizol LS (Invitrogen). cDNA was synthesized by using Superscript II reverse transcriptase (Invitrogen) according to the manufacturer's recommendations. The PCR primers are available upon request.

### Flow cytometry

Antibodies were prepared at the concentration of 0.1 mg/mL. 10 μL of the stock solution was added to 1 – 2.5 × 10^5 ^cells in a total reaction volume not exceeding 200 μL. The sample was then incubated for 20 min at 2–8 °C. Following incubation, excess antibody was removed by washing cells twice with FACS buffer (2% FCS and 0.1% sodium azide in Hank's buffer). After wash, cells were resuspend in 200 μL of FACS buffer and the binding of unlabeled monoclonal antibodies was visualized by adding 10 μL of a 25 μg/mL stock solution of a secondary developing reagent such as goat anti-mouse IgG conjugated to a fluorochrome for 20 min at 2–8°C. Following incubation, cells were washed once with FACS buffer, once with PBS. After wash, cells were resuspend in 400 μL of PBS and analyzed on a FACScant flow cytometer (Becton-Dickinson, Mountain View, CA). Five thousand events were collected and analyzed using CELL Quest software.

## Authors' contributions

Dr. Cai contributed significantly in validating antibodies in human ES cells and human EBs. Ms. Olson performed initial screening of antibodies in various cell lines. Dr. Rao initiated the project, supervised Dr. Cai, and participated in all discussions for this report. Ms. Stanley and Ms. Taylor performed Western blot analysis. Dr. Ni coordinated collaborative work between two labs, monitored the generation of the antibodies, and directed the project at R&D Systems.
